# Plumes from Using Iron to Boil Liquid Nitrogen to Illustrate the Importance of Surface Area

**DOI:** 10.1021/acs.jchemed.2c00699

**Published:** 2023-03-14

**Authors:** Dean J. Campbell, Thomas S. Kuntzleman, Kayla Lippincott, Abe Yassin, Khitab Dar, Q Ott

**Affiliations:** † Mund-Lagowski Department of Chemistry and Biochemistry, Bradley University, Peoria, Illinois 61625, United States; ‡ Department of Chemistry, Spring Arbor University, Spring Arbor, Michigan 49283, United States

**Keywords:** General Public, First-Year Undergraduate/General, Demonstrations, Analogies/Transfer, Kinetics

## Abstract

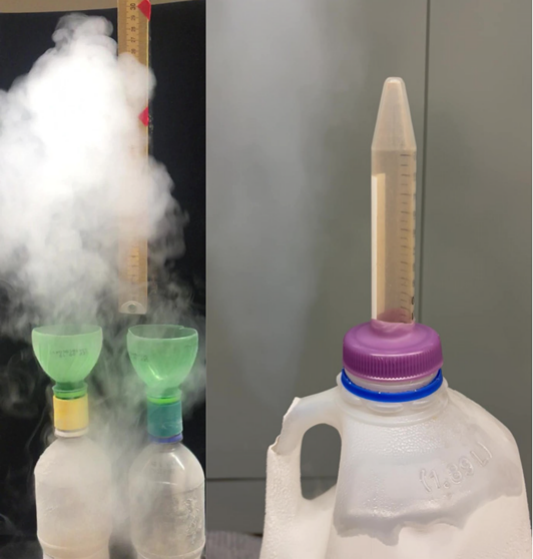

The relationship between surface area and dynamics of processes can be demonstrated by adding iron at room temperature to liquid nitrogen. The rate at which the liquid nitrogen boils to produce gas is related to the surface area of the iron. Adding iron in the form of consistent units that have measurable sizes can be readily connected to observable differences in rates of nitrogen gas production. For example, samples of smaller iron spheres with their greater surface area transfer heat more quickly than do larger spheres of the same volume to liquid nitrogen causing it to boil faster, but more briefly, and produce larger plumes of nitrogen gas from a container vent. The plumes are essentially comprised of nitrogen and water, which make them potentially safer than plumes from other demonstrations such as the “genie in a bottle”, based on hydrogen peroxide decomposition. These simple activities can be used as stand-alone demonstrations or as the basis of laboratory activities.

## Introduction

Many heterogeneous chemical and physical processes are surface-area dependent; that is, the processes proceed more quickly when there is increased contact between reactive phases. For example, soluble solids will dissolve in a liquid more quickly when they are crushed into a powder with a high surface area. A number of demonstrations have been published in this *Journal* illustrating this concept.^1−3^ This paper describes ways these ideas can be explored by using iron objects (e.g., iron or steel spheres) of varying sizes in contact with liquid nitrogen.

The conversion of liquid nitrogen to gaseous nitrogen is an endothermic process:
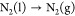
1

Given that this process requires energy, it is reasonable to assume that the rate of liquid nitrogen conversion to gas will increase as the rate of thermal energy transfer into the liquid nitrogen increases. This relationship between rate of thermal energy transfer and rate of conversion to gas can be observed by adding iron spheres under various conditions into liquid nitrogen and observing the condensation plumes produced. Given two samples of iron spheres of the same mass added to liquid nitrogen, the sample with the smaller spheres will have a greater area of surface in contact with the liquid. As a result, the smaller sphere samples will be able to transfer heat into the liquid nitrogen more quickly, causing it to boil more vigorously (but over a shorter period of time) to produce larger plumes of nitrogen gas.

These demonstrations can be used as alternative plume-producing demonstrations in place of the catalytic “genie in the bottle”.^4^ In the well-known “genie” demonstration, a catalyst such as potassium iodide or manganese(IV) oxide is added to 30% hydrogen peroxide to produce heat and raise a plume of oxygen gas, water, and other substances in the air. Safety concerns regarding demonstrators and audience members inhaling catalyst, unreacted peroxide, and other substances have led to a moratorium on this demonstration at this University.^5^ A plume comprised of nitrogen gas and water droplets can be considered to be safer by comparison. Although the boiling of liquid nitrogen is not a catalyzed process like the peroxide demonstration, it still can be used to demonstrate the impact of surface area on processes, and heterogeneous catalysis is indeed accelerated by increased surface area.

## Experimental Section

### Iron Spheres and Other Components

Iron was selected for this work because it is very Earth-abundant, has low toxicity, can withstand cooling in liquid nitrogen without mechanical failure or chemical reaction, and has a high thermal conductivity. The iron spheres can be used multiple times, improving the sustainability of these activities. Iron is ferromagnetic, enabling its movement into, within, and out of lab equipment to be controlled by magnets.^6^

Table 1 describes the iron spheres used in these studies. The steel spheres were obtained from various sources (e.g., BC Precision, Chattanooga, TN; https://bcprecision.business.site/) and did not have any additional metal cladding. The BB iron shot (BBs) was obtained from the Crosman Corp., Bloomfield, NY (https://www.crosman.com/), and was composed of iron spheres with copper cladding. A variety of approaches were attempted to remove the copper cladding to initially produce all-iron surfaces. It appeared that the most effective way to remove the cladding from the BBs and/or roughen the surface of all of the spheres was to tumble the spheres in a rock tumbler with water and sand. The iron filings were obtained from the Fisher Scientific Co., Fair Lawn, NJ, now with the website https://www.thermofisher.com/.

**Table 1:** Iron Spheres Used in the Activities

nominal size of iron spheres	diameter/mm	surface area/mm^2^	number of spheres to make ∼8.3 g
half-inch spheres	12.7	507	1
quarter-inch spheres	6.4	129	8
BBs (originally copper clad)	4.3	58	25
eighth-inch spheres	3.2	32	64
40 mesh iron filings^a^	0.42	0.55^a^	27000

a The iron filings were not all uniform in size and shape but here are approximated as spheres with diameters corresponding to the hole sizes of a 40 mesh sieve.

The iron spheres do not need to be flawlessly clean to achieve success in these liquid nitrogen experiments, as heat from the spheres will boil the nitrogen whether their surfaces are rusty or not. In our planet’s oxidizing atmosphere, the iron spheres will eventually form rust on their surfaces (even copper-clad iron BBs will rust), so it is probably easiest to leave them rusty.

Other components of these activities can be also considered in the context of the safety to people and the environment.^7^ Elemental nitrogen is Earth abundant and has low toxicity. The liquid nitrogen used in these experiments is essentially borrowed from the atmosphere as a gas, liquefied, and returned to the atmosphere as a gas during the course of the demonstrations. There are safety risks in working with liquid nitrogen; see the Safety section.^8,9^ The plastic bottles and jugs were repurposed from their use as beverage containers.

### Boiling Liquid Nitrogen to Produce Gas Plumes from Soda Bottles

**Figure 1:**
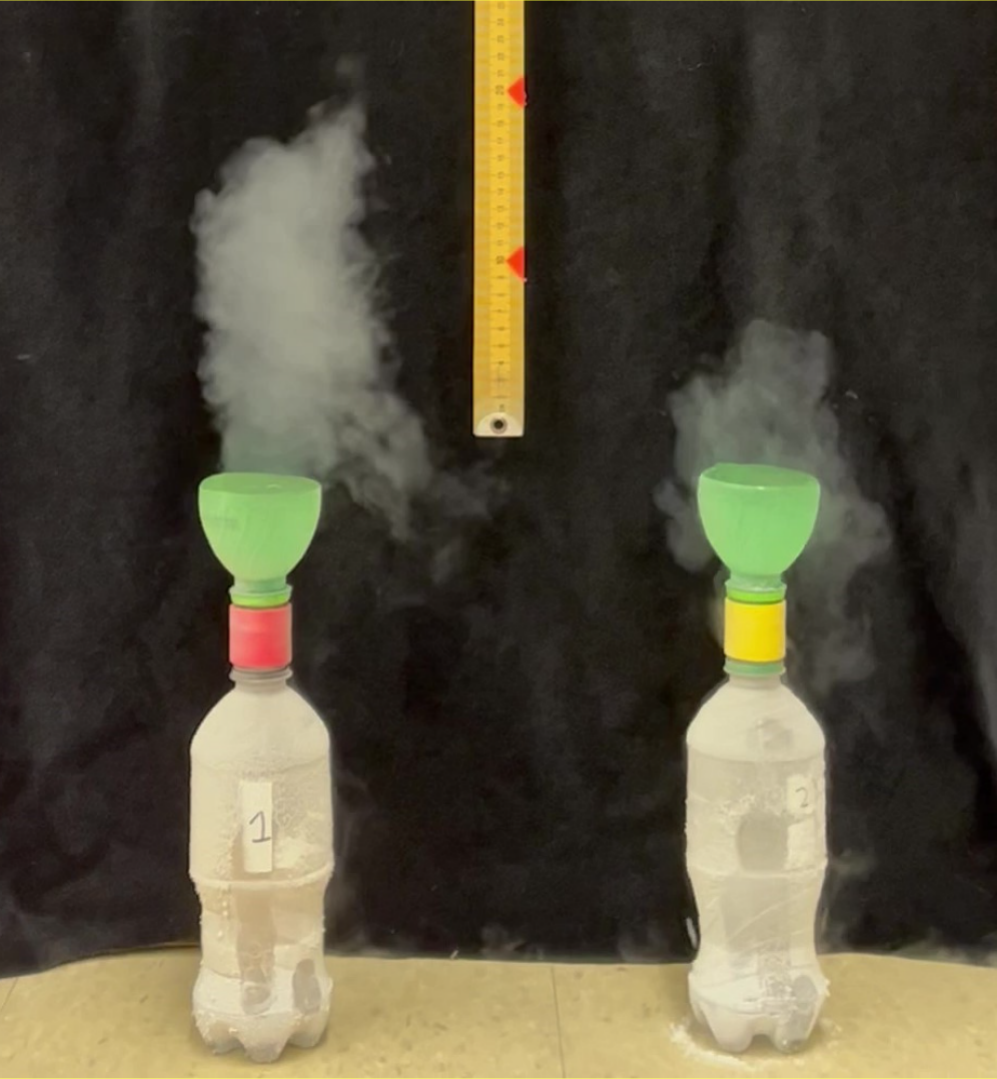
Fifty grams of iron spheres added to 250 mL of liquid nitrogen in 500 mL soda bottles at the same time, with rusty BBs on the left and half-inch rusty spheres on the right. The plume on the left rises higher but fades more quickly than the one on the right.

In one set of demonstrations, iron spheres at room temperature were added to plastic bottles containing liquid nitrogen, and the rates of boiling the nitrogen were observed. Six half-inch iron spheres with 3000 mm^2^ total surface area and about 140 BBs with 8600 mm^2^ total surface area were weighed in plastic weigh boats. Each sample had a total mass of about 50 g. Two dry, empty 500 mL plastic soda bottles were placed in an upright position. They were secured with 3-finger clamps to a ring stand. The liquid nitrogen was added via a variety of funnels, described further in the Supporting Information. The bottles were filled with approximately 250 mL of liquid nitrogen. The spheres were then quickly poured from the weigh boats into the same funnels to add to the liquid nitrogen already in the bottles. In any of these setups, the sphere samples were dropped into their respective bottles at the same time, and the resulting water condensation plumes were observed (Figure 1). In all cases, the plumes initially rose and faded more quickly for the smaller spheres. By contrast, the plumes for the larger spheres rose less, but faded more slowly.

The nitrogen gas produced in this manner was sufficient to inflate and burst a 9-in. diameter balloon placed over the end of a bottle. This effect was used to demonstrate the effect of increased surface area on thermal energy transfer: iron spheres were placed into a balloon, the balloon’s mouth was quickly placed over the end of the bottle, and the spheres were dropped into the liquid nitrogen. 50 g of BBs inflated and popped a balloon faster than did 50 g of half-inch iron spheres.

### Boiling Liquid Nitrogen to Produce Gas Plumes from a Single Plastic Milk Jug

**Figure 2:**
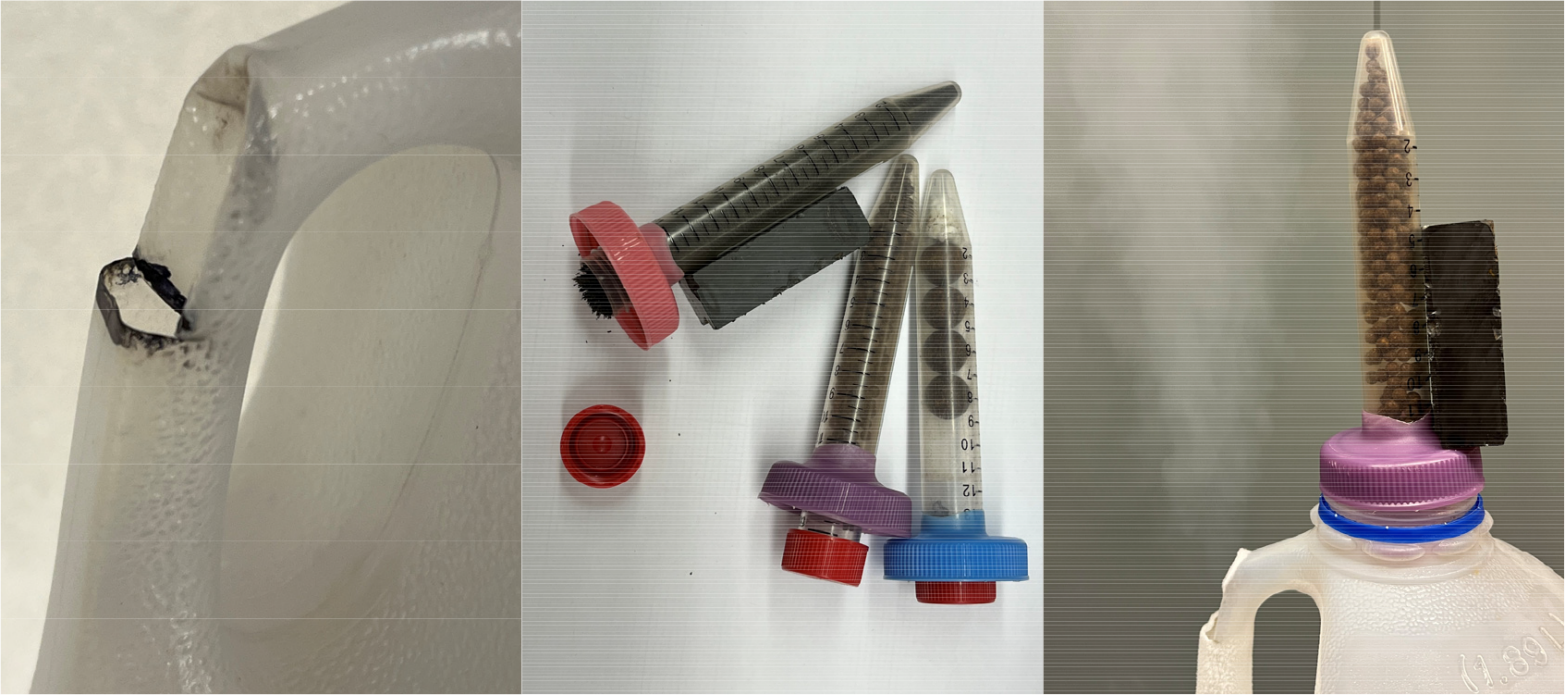
(Left) Close-up of the nitrogen gas vent made in the plastic milk jug handle. (Middle) Plastic 15 mL centrifuge tubes inserted into plastic milk jug caps and filled with iron filings, eighth-inch iron spheres, and half-inch iron spheres. (Right) A centrifuge tube/milk cap assembly with iron spheres and a magnet screwed onto the main opening of a plastic milk jug. Note the condensation plume coming from the vent.

A more-recently developed demonstration to show plume output in a sequential, rather than side-by-side format used a half-gallon (2 L) plastic milk jug. The assembly is a bit more complex, but produced much larger plumes. The plume was produced from a vent in the upper part of the handle of the jug. To make the vent, a slit was cut in the handle, and part was pushed in to make the hole shown in Figure 2 (left). For each plastic milk jug screw cap, a hole was made, and a 15 mL plastic centrifuge tube was inserted from the threaded side of the cap (Figure 2, middle). The iron was then placed into the centrifuge tube and held in place with a magnet until the iron was dropped into the liquid nitrogen. For longer-term containment or storage of the iron in the tube, a centrifuge tube cap was screwed onto the tube opening. A little bit of padding such as polystyrene foam was placed in the end of the interior of the tube to prevent the iron from breaking the end of the tube or getting stuck inside. Multiple tube assemblies containing iron were used with one milk jug containing liquid nitrogen. Figure 2 (right) shows a centrifuge tube/milk cap assembly loaded with iron spheres held in place with a magnet. The cap was screwed onto the main opening of the milk jug.

**Figure 3:**
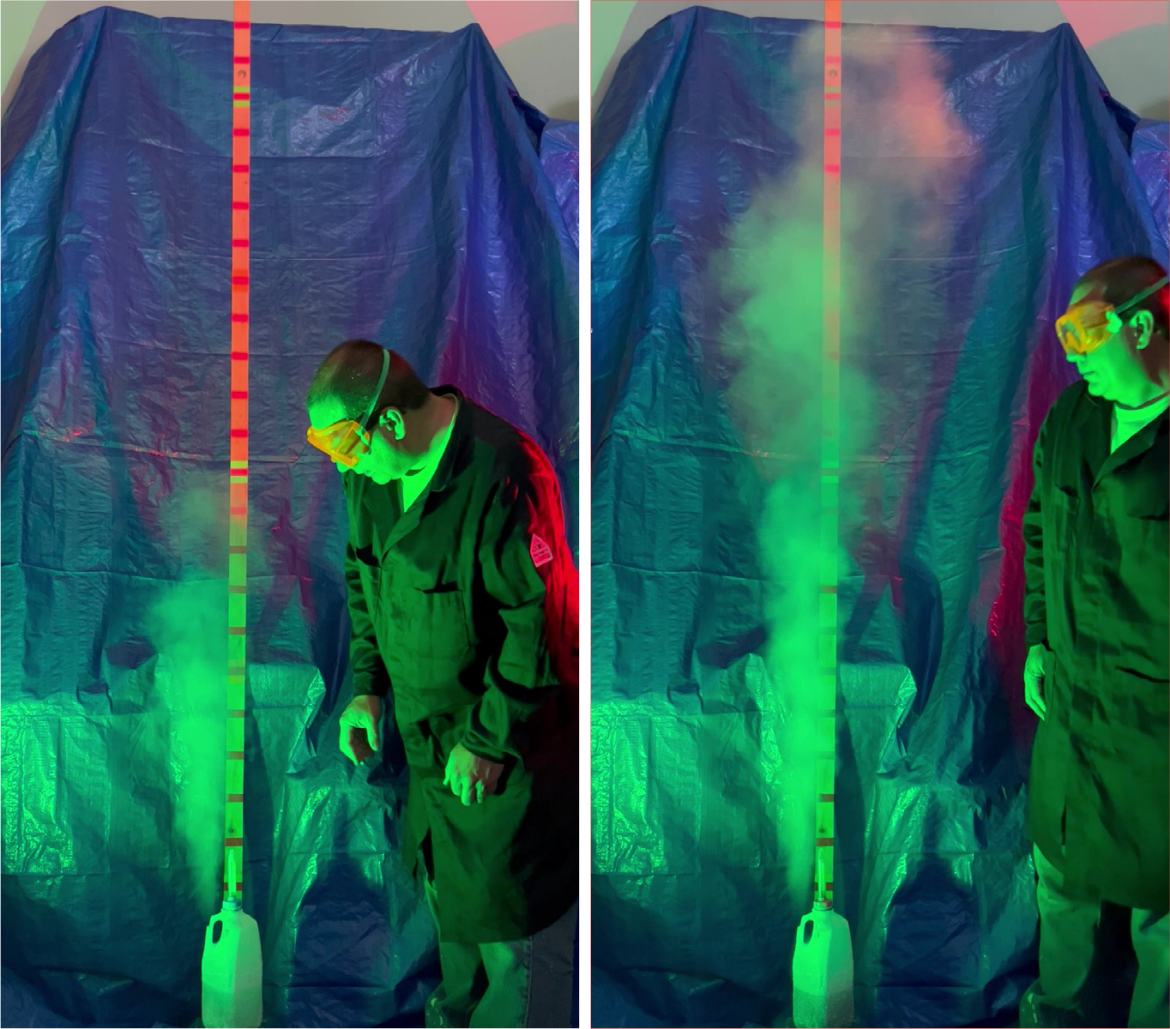
Nitrogen gas/water condensation plumes produced by adding about 33 g of iron to liquid nitrogen in the form of (left) four half-inch iron spheres and (right) iron filings.

Using these assemblies, the plumes produced by adding 33 g of half-inch iron spheres were compared to those produced by 33 g of iron powder to jugs filled about half full with liquid nitrogen. It should be noted that the iron was not added until enough time had passed for the rapid boiling of the liquid to cease after adding it to the jugs. Audience members were shown a rectangle of graph paper that had the same surface area as the four half-inch iron spheres (2000 mm^2^). The assembly with those spheres then was connected to the jug, and the magnet was removed. The spheres dropped into the liquid nitrogen, causing it to produce cold nitrogen gas, which vented out of the bottle to become a plume that reached a height of about a meter (Figure 3, left). A measuring stick marked in 10 cm intervals was placed behind the plumes. The plume was allowed to shrink back down in size as the iron cooled before more iron was added. Next, audience members were shown a rectangle of graph paper that had the same surface area as 33 g of the iron filings (59 000 mm^2^). The assembly with the filings and a magnet then was connected to the jug, and the magnet was removed. The filings dropped into the liquid nitrogen, causing it to quickly produce nitrogen gas, which vented out of the bottle to become a plume that reached a height that was well more than 2 m tall (Figure 3, right). This larger plume lasted for less time than the smaller plume. A video of this demonstration in action can be viewed at https://www.youtube.com/watch?v=05MwuO2JAKE.

**Figure 4:**
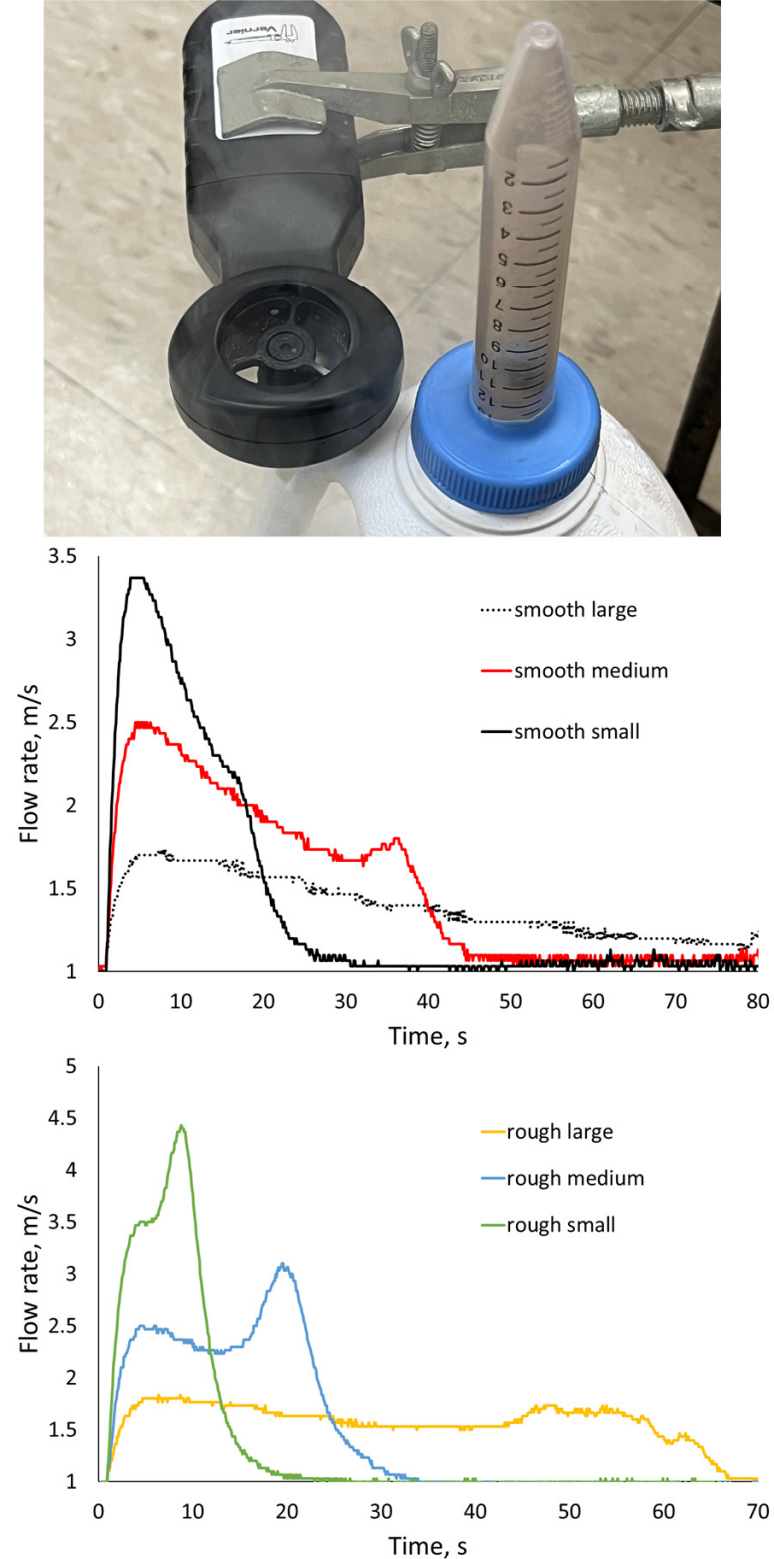
(Top) Vernier anemometer placed over the vent of a liquid nitrogen-containing bottle to measure its nitrogen output flow rate. (Middle) Graphs of flow rate as a function of time for smooth half-, quarter-, and eighth-inch iron spheres added (average of three trials). (Bottom) Graphs of flow rate as a function of time for rusty half-, quarter-, and eighth-inch iron spheres added (average of three trials).

Figure 4 shows the intriguing results of experiments where a Vernier anemometer was used to quantify the flow of nitrogen vapor leaving the bottles containing liquid nitrogen. Samples (∼33 g masses) composed of smaller spheres produced plumes that had higher peak velocities, but lasted for less time, than samples composed of larger spheres. Rusty iron spheres produced plumes that had higher peak velocities than samples composed of smooth spheres of the same size. Perhaps the rough surfaces of the rusty spheres had more nucleation sites for forming nitrogen gas bubbles as the liquid nitrogen boiled.

## Safety

Plastic can become brittle at low temperature. Make sure all containers that contain liquid nitrogen are properly vented; never seal up a container with liquid nitrogen inside.^8,9^ Proper personal protective equipment such as goggles must be used, especially considering vertical plumes of materials are produced from potentially cold-embrittled materials. Avoid spilling reagents on clothing. Avoid skin contact and wear insulating gloves while working with the liquid nitrogen or working with objects that have been cooled by liquid nitrogen. Always wash hands after completing the demonstrations.

## Discussion

We have used these nitrogen plume-producing activities in various outreach events to demonstrate that the amount of surface area of contact between iron and liquid nitrogen can very much impact the rate of boiling. Smaller spheres produce larger, but shorter-lived, plumes. Because viewers in an audience cannot necessarily see the individual iron objects used in these experiments, it is important to describe the cumulative surface area of the iron in the samples. This can be done by holding up appropriately sized pieces of graph paper, or even by using hand gesture estimates. It is important to point out that even though the rate of liquid nitrogen boiling changes, the amount of liquid nitrogen that boils does not change, because the same amount of heat was transferred from the same masses of metal, regardless of surface area.

Despite the large ruler shown in Figure 3, no attempt was made to precisely quantify the size of the plumes. The plumes were often produced in attempts to maximize their visibility, using strategies such as lights illuminating the plume from the side and a dark background. However, in classrooms and outreach events, there might be less control over such factors. Air currents in the demonstration space and humidity have also been observed to impact plume visibility. For example, humid air tends to produce more opaque plumes.^10^ In almost all cases, using two or three samples of iron objects with very different sizes is sufficient to illustrate surface area effects. For example, in a recent outreach event featuring audience members ranging from middle school through adults, we successfully compared plumes from the milk jug for three 25 g iron samples: half-inch spheres, eighth-inch spheres, and iron filings. The plumes correlated with the surface area: the half-inch spheres created the smallest plume, the iron filings produced the largest plume, and the eighth-inch spheres produced a plume of intermediate height. In a thank you card received from the audience members, there was mention of this demonstration: “When you added the liquid nitrogen and metal pellets and powder together, we were amazed at how high it shot up.”

## Supporting Information Available

Measurement of the heat capacity of the rusty iron spheres as they boil liquid nitrogen, and other activities described in this work (PDF, DOCX)
