# Supplementary material for: Plumes from Using Iron to Boil Liquid Nitrogen to Illustrate the Importance of Surface Area
Source: J Chem Educ. 2023 Mar 14;100(4):1699–703. doi: 10.1021/acs.jchemed.2c00699 (PMC10100552; doi:10.1021/acs.jchemed.2c00699)
Supplement: Supplementary file 1 — ed2c00699_si_001.pdf [file ed2c00699_si_001.pdf]

Supporting Information:

Plumes from Using Iron to Boil Liquid Nitrogen to Illustrate the Importance of Surface Area

Dean J. Campbell*‡, Thomas S. Kuntzleman**, Kayla Lippincott*, Abe Yassin*, Khitab Dar*, Q Ott*

*Mund-Lagowski Department of Chemistry and Biochemistry, Bradley University, Peoria, Illinois 61625, United States

**Department of Chemistry, Spring Arbor University, Spring Arbor, Michigan 49283, United States

‡ Email: campbell@fsmail.bradley.edu

Safety warning

All reagent containers should be clearly labeled. Proper personal protective equipment such as goggles should be used, ESPECIALLY considering vertically rising foams and vapor plumes can be produced. Avoid spilling reagents on clothing. Avoid skin contact and wear insulating gloves while working with the liquid nitrogen or working with objects that have been cooled by liquid nitrogen. Always wash your hands after completing the activities.

Funnels/connector assemblies for dropping rusty iron spheres into bottles

Funnels can be made from 500 mL soda bottles, and then connected to the lower bottle with a drill-widened tornado tube or a ½-inch (12.7 mm) outer diameter PVC tubing slid into the inside of the bottle openings. Figure S1 shows funnels and connector assemblies for dropping iron spheres into bottles using tornado tubes and PVC pipes. The LEFT picture shows an unaltered tornado tube connector and a bottle top funnel connected to a drilled-out tornado tube connector connected to a 500 mL bottle. The RIGHT picture shows a half-inch outer diameter PVC tube with outside edges beveled to slide the tube into the bottle ends. The inside edges are also beveled to allow the beads to easily enter the tube. The picture also shows the bottle top funnel connected to a beveled PVC tube connector connected to a 500 mL bottle. In still another approach, the liquid nitrogen was added to each empty bottle using an

unattached, smaller-mouth funnel that was removed after the fill. Spheres could also be added to the bottles using soda fountain launchers (e.g., Geyser Tube, Steve Spangler Science, Littleton, CO). When doing so, the iron spheres were held in place inside of the Geyser Tube by a magnet placed outside of the tube. The loaded soda fountain launchers were quickly screwed onto the openings of the soda bottles, being careful not to let the spheres fall prematurely into the liquid nitrogen. Figure S2 shows the PVC tubes in use. It should be noted that shorter PVC tubes might also make effective funnels.

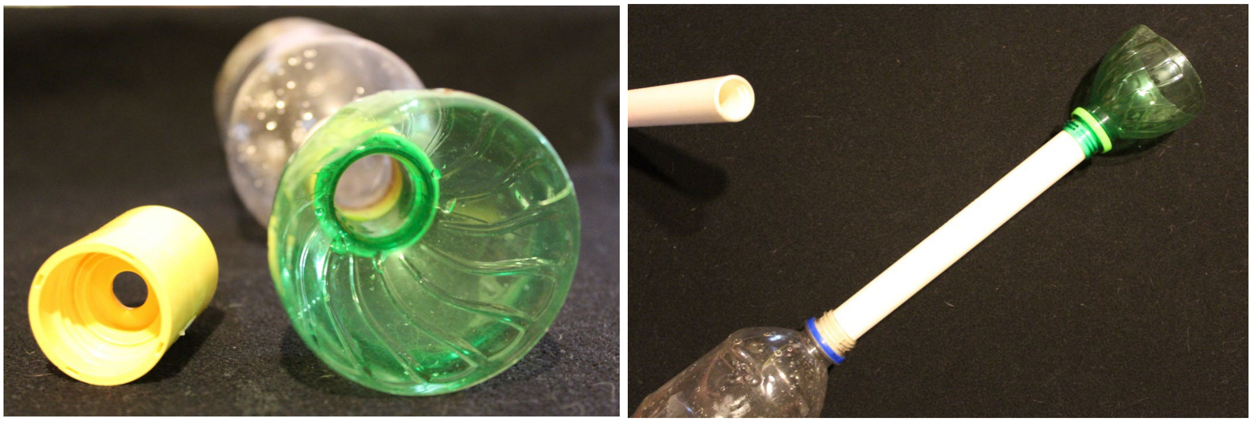

Figure S1. (LEFT PICTURE) LEFT: Tornado tube connector. RIGHT: Bottle top funnel connected to drilled-out tornado tube connector connected to a 500 mL bottle. (RIGHT PICTURE) LEFT: Half-inch outer diameter PVC tube with outside edges beveled to slide the tube into the bottle ends. The inside edges are also beveled to allow the beads to easily enter the tube. RIGHT: Bottle top funnel connected to beveled PVC tube connector connected to a 500 mL bottle.

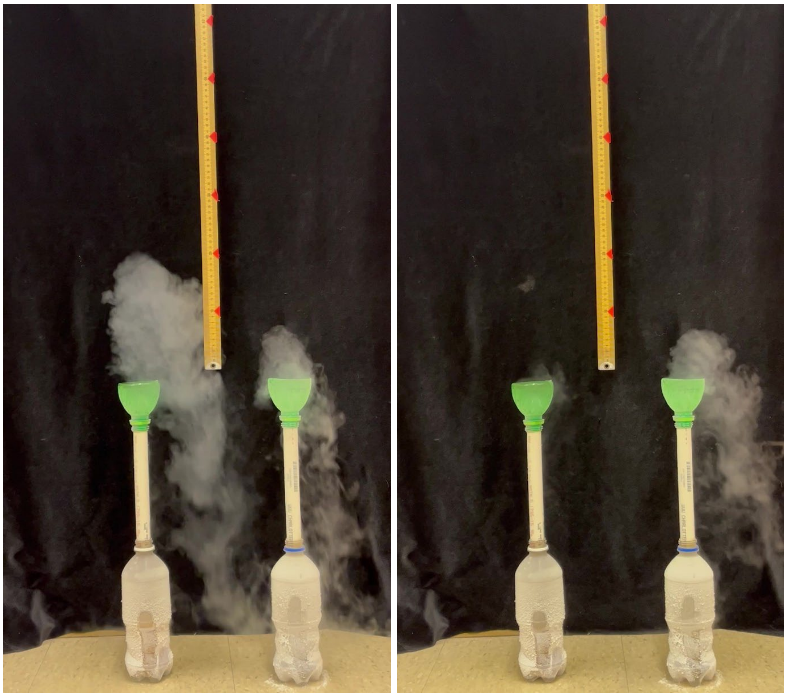

Figure S2. 50 g of iron spheres added to 250 mL of liquid nitrogen in 500 mL soda bottles at the same time, with rusty BBs at left and half-inch rusty spheres at right. The plume at left rises higher but fades more quickly than the one at right. (LEFT) Approximately 8 seconds after addition of spheres. (RIGHT) Approximately 18 seconds after addition of spheres.
